# Plasma Supported Deposition of Amorphous Hydrogenated Carbon (a-C:H) on Polyamide 6: Determining Interlayer Completion and Dehydrogenation Effects during Layer Growth

**DOI:** 10.3390/polym13111886

**Published:** 2021-06-06

**Authors:** Torben Schlebrowski, Henriette Lüber, Lucas Beucher, Melanie Fritz, Youssef Benjillali, Mohammed Bentaouit, Barbara Hahn, Stefan Wehner, Christian B. Fischer

**Affiliations:** 1 Department of Physics, University Koblenz-Landau, 56070 Koblenz, Germany; hlueber@uni-koblenz.de (H.L.); lucasbeucher@uni-koblenz.de (L.B.); melfritz@uni-koblenz.de (M.F.); wehner@uni-koblenz.de (S.W.); 2 Department of Material Analysis, RheinAhrCampus, University of Applied Sciences, 53424 Remagen, Germany; youssef-ben@live.de (Y.B.); mbentaou@rheinahrcampus.de (M.B.); barbara-m-hahn@t-online.de (B.H.); 3 Materials Science, Energy and Nano-Engineering Department, Mohammed VI Polytechnic University, Ben Guerir 43150, Morocco

**Keywords:** PECVD plasma coating, gradual film deposition, synchrotron radiation, wettability, a-C:H polymer interlayer

## Abstract

Polyamide 6 (PA6) is a commonly used material in many different sectors of modern industry. Herein, PA6 samples were coated with amorphous carbon layers (a-C:H) with increasing thickness up to 2 µm using radio frequency plasma enhanced chemical vapor deposition for surface adjustment. The morphology of the carbon coatings was inspected by ex situ atomic force microscopy and scanning electron microscopy. Surface wettability was checked by contact angle measurements. The chemical composition was analyzed using the surface sensitive synchrotron X-ray-based techniques near-edge X-ray absorption fine structure and X-ray photoelectron spectroscopy, supported by diffuse reflectance infrared Fourier transform spectroscopy. Particular attention was paid to the coating interval from 0 to 100 nm, to specify the interlayer thickness between the PA6 polymer and a-C:H coating, and the region between 1000 and 2000 nm, where dehydrogenation of the a-C:H layer occurs. The interlayer is decisive for the linkage of the deposited carbon layer on the polymer: the more pronounced it is, the better the adhesion. The thickness of the interlayer could be narrowed down to 40 nm in all used methods, and the dehydrogenation process takes place at a layer thickness of 1500 nm.

## 1. Introduction

Polyamide polymers are widely used in many aspects of modern life such as mechanical, electrical, and sanitary engineering [1], in the automotive [2] and construction industries, and in medical technology, e.g., for artificial joints [3], surgical instruments, and as suture material [1]. Additionally, polyamide 6 (PA6) is used for food and medical packaging [4] and for composite materials [5,6]. Nevertheless, probably the best known application of polyamides is their use as nylon for clothes and ropes [7]. Polymers (including polyamides) have in common great advantages (elasticity, formability, low weight, and good usability in a wide range of temperatures as well as chemical resistance) [8,9], but their applicability is limited due to, e.g., low hardness, low abrasion resistance, or poor mechanical properties [8].

One possible way to adapt polymers for further applications is to perform comprehensive surface modification by the deposition of hydrogenated amorphous carbon [10,11,12,13,14,15,16]. Although polyamides are widely used in their application, only little research has been done on surface modification with such coatings [17]. The films deposited mainly consist of carbon and contain sp^2^ (π and σ) and sp^3^ bonding sites with hydrogen incorporated in the network [11,18,19]. Depending on the dominant bond, such layers can have diamond-like properties (high hardness, chemical inertness, high electrical resistance) for a low sp^2^/sp^3^ ratio or more graphite-like characteristics (softer layer, improved electrical conductivity) if the sp^2^/sp^3^ ratio is increased [20]. Which bonding site dominates can be controlled with the help of a few parameters such as the plasma conditions, precursor gas, amount of hydrogen [11,19,20,21,22], or the layer thickness itself [23,24,25,26]. It should also be noted that this change in the chemical composition of the coating has a direct influence on the wettability of the surface [27].

For these layers, however, the strength of their connection with the base material is important. If this is not sufficient, the layer can be easily detached from the material. The more pronounced the interlayer, the better the bond. The interlayer is a mixed phase of base polymer and the deposited layer [28]. The thickness varies between a few nm (e.g., POM), which results in only a low degree of binding and easy layer removal, and several tens of nm as for HDPE, leading to a strong connection of the layer with the base material [14,16,23]. Additionally, for thicker layers, the effect of dehydrogenation occurs. Here, hydrogen is removed from the layer and the content of sp^3^-bonded carbon is reduced in favor of increasing sp^2^ content [14,15,16].

These layers can be deposited by a wide variety of techniques, including magnetron sputtering, ion beam deposition or, as in the case of the films analyzed in this work, by means of radio frequency plasma enhanced chemical vapor deposition (RF-PECVD) [11,14,20,28]. A major advantage is that this technique can also be used to coat polymeric substrates, which are usually non-conductive and require low process temperatures [13,20]

In the present study, a-C:H layers of variable thickness were deposited on the polymer polyamide 6 (PA6). This was realized by RF-PECVD technique with an acetylene plasma. The surface morphology of these deposited carbon layers was investigated ex situ by atomic force microscopy (AFM) and scanning electron microscopy (SEM). Diffusive reflectance infrared Fourier transform (DRIFT) and surface sensitive X-ray-based techniques such as near-edge X-ray absorption fine structure (NEXAFS) and X-ray photoelectron spectroscopy (XPS) were used to study the chemical composition of the deposited layers. In addition, contact angle (CA) measurements were performed to evaluate the relationship between sp^2^/sp^3^ ratios and macroscopic physical aspects.

## 2. Materials and Methods

### 2.1. Sample Preparation and Coating Process

The polyamide 6 plates (thickness 1 mm) used in this study were delivered by Goodfellow (Bad Nauheim, Germany) and of industrial quality. Stamped samples of PA6 (Ø 10 mm) were ultrasonically cleaned with isopropanol, dried at ambient conditions, attached on an aluminum sample holder, and positioned in front of the plasma source (fixed distance 275 mm). The generation of the plasma in a vacuum chamber is realized with a high-frequency (RF, 13.65 MHz) plasma source (Copra DN 400, CCR GmbH, Troisdorf, Germany) [28]. The coating process itself is described in detail elsewhere [26]. In brief: First, there was a 10 min pretreatment with O_2_ plasma (sample cleaning and surface activation). Second, exposure to C_2_H_2_ plasma for a-C:H layer deposition, where the thickness can be controlled by varying the coating time [14,15,16,29]. During the entire plasma process, the temperature of the samples did not exceed 40° at any time [29]. Thereby, a-C:H layer thicknesses from 0 nm (O_2_-treated sample) up to 2000 nm (deposition rate 10 nm/min [29]) were generated. Below a layer thickness of 100 nm, coatings were realized in 10 nm steps to analyze the initial a-C:H layer growth. For higher thicknesses, steps of 50, 100, and finally 250 nm between 1000 to 2000 nm were chosen. To determine the deposited a-C:H thickness with a profilometer (Dektak 3, Veeco Instruments Inc., Plainview, NY, USA), half covered silicon wafers were used [26].

### 2.2. Surface Morphology

The surface morphology was identified using scanning electron microscopy (SEM515, Phillips, 7 kV, WD 20 mm, FEI Company, Amsterdam, The Netherlands) for a more macroscopic overview and atomic force microscopy (AFM, Omicron nanoTechnology GmbH, Taunusstein, Germany) for a detailed mapping. For SEM it was necessary to apply gold (7–10 nm thick) to the samples to increase their conductivity and thus protect them from charging effects and electron beam damage. Both the homogeneity of the sample surfaces and the accuracy of the measurements were provided by recording at least three distinct surface sites. The AFM was operated in contact mode with standard silicon nitride PNP-TR cantilevers (NanoAndMore GmbH, Wetzlar, Germany) at ambient conditions. Cellulose acetate replica (Pelco, 607-AFM) with a 2.160 lines/mm waffle pattern diffraction grating were measured regularly to avoid tip sample convolution and tip blending. Recordings at three different sites ensured that the measurements were performed correctly and that no image errors occurred. Image analysis (SPIP version 4.6.1, Image Metrology A/S, Hørsholm, Denmark) was conducted according to the following steps: LMS fit of grade 4 for plane-correction; a median filter for reducing the worst horizontal noise; reduction of the low frequency noise by using a convolution smooth low pass filter and a convolution smooth mean filter; removing long waves and filtering out values outside of the color boundaries [29]. To investigate the sample surface wettability, contact angle (CA) measurements were performed using an OCA15EC (Dataphysics Instruments GmbH, Filderstadt, Germany) at ambient conditions: sessile drop technique, 1 µL water (HPLC grade, CHEMSOLUTE^®^, Th. Geyer GmbH & Co. KG, Renningen, Germany) at five different sites, and resulting CA averaging. This way, the homogeneity of the sample surface could be additionally checked.

### 2.3. Infrared Spectroscopy

For the chemical composition of each sample surface, diffuse reflectance infrared Fourier transform (DRIFT) measurements were performed at ambient conditions. For this purpose, a Shimadzu Fourier transform spectrometer (IRPrestige-21, Shimadzu Corporation, Kyoto, Japan) equipped with the diffuse reflectance measuring apparatus DRS-8000 was used [30,31]. Measurements were repeated at three different surface sites to ensure surface homogeneity. For each sample, two spectra were recorded: a spectral survey (500–4000 cm^−1^ resolution: 4 wavenumbers, 100 repetitions) followed by a detailed recording for the C–H stretching region [26,32] (2800–3100 cm^−1^ resolution: 1 wavenumber, 300 repetitions). The overall reference for the a-C:H data analysis is the O_2_ plasma-cleaned PA6 sample, as each one has been O_2_ plasma pretreated. FTIR Control Software (software version 1.30, Shimadzu Corporation, Kyoto, Japan) was used for a two-step spectral evaluation: a multipoint baseline insertion with the integrated manipulation tool and afterwards a smoothing manipulation.

### 2.4. X-ray Spectroscopy

The X-ray photoelectron spectroscopy (XPS) and near-edge X-ray absorption fine structure (NEXAFS) measurements were performed at the synchrotron BESSY, Helmholtz-Zentrum Berlin, during the low-alpha phase and used as supportive studies of the carbon bond state on the surface. The used HE-SGM beamline system is described elsewhere [33]. To prevent charge-induced effects on the sample surface it is additionally equipped with a flood gun.

During the XPS measurements, a full spectrum (700 to 0 eV) was first recorded. This survey provides an overview of the entire chemical surface composition. The C1s peak was examined to analyze the different binding states of the carbon atoms. These measurements were performed at least at two different sample sites to check reproducibility, homogeneity, and the stability of the deposited a-C:H layers. For the analysis of the received spectra and the deconvolution of the C1s spectra, the commercially available software CasaXPS (software version 2.3.18, Casa Software Ltd., Teignmouth, UK) was used. The percentages of sp^2^, sp^3^, C–O, and C=O bonds in the total C1s peak were identified, where C–O bonds were evaluated as sp^3^ bond and the C=O bond as sp^2^-bonded carbon, and related to the corresponding thicknesses. To compensate for static charges, the scale of the binding energy was corrected in the evaluations. The C1s peak was set to a value of 284.8 eV [33].

The C as well as the O K-edges were recorded with NEXAFS. For the C K edge, measurements were taken on at least two different surface sites to ensure accuracy. O K-edges were recorded to check the presence or absence of oxygen. All measurements were performed in partial electron yield (PEY) in which a counter voltage is applied. In this way, the measurement electronics are not reached by all the electrons leaving the material, meaning it is very surface sensitive [34]. Detector and sample geometry followed an incidence angle of 55°. The C K-edges were analyzed using Origin 8.1 commercial software. For the analysis according to Watts et al. [35], it was necessary to first normalize the spectra and then adjust them to the decreasing ring current present at the BESSY experimental station. Subsequently, a correction with a previously measured gold edge was performed to eliminate a possible contamination of the grid. Further analysis was performed using a self-written peak evaluation program combined with Origin 8.1 software.

## 3. Results

### 3.1. Surface Morphology

**Figure 1:**
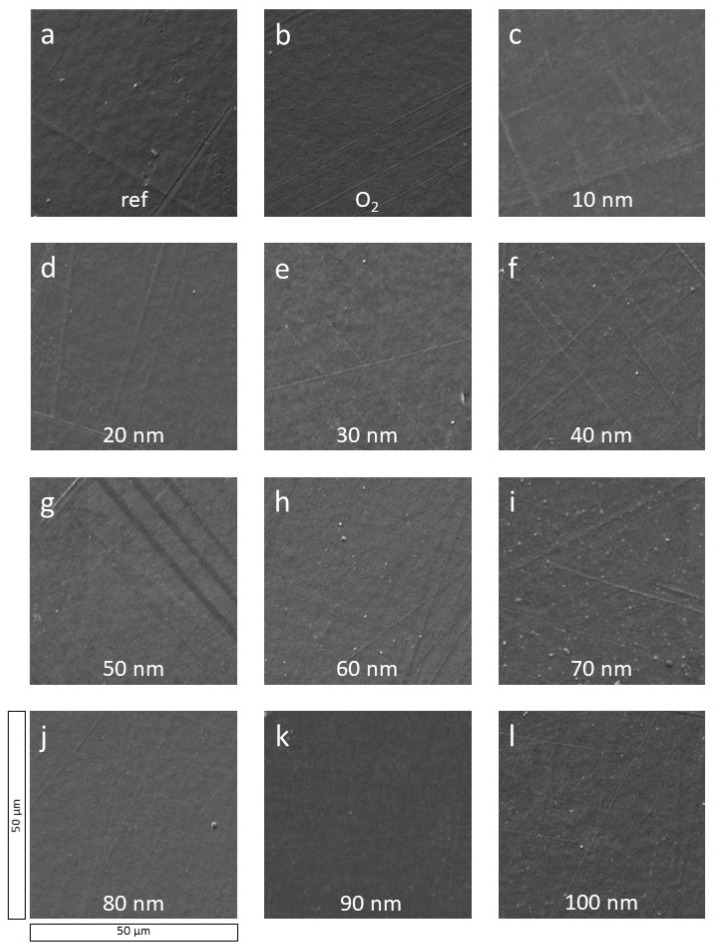
SEM surface images of PA6 samples coated with thin a-C:H layers from 10 to 100 nm in 10 nm steps (**c**–**l**). Additionally, the reference (**a**) and the O_2_ plasma-treated PA6 sample (**b**) are added.

**Figure 2:**
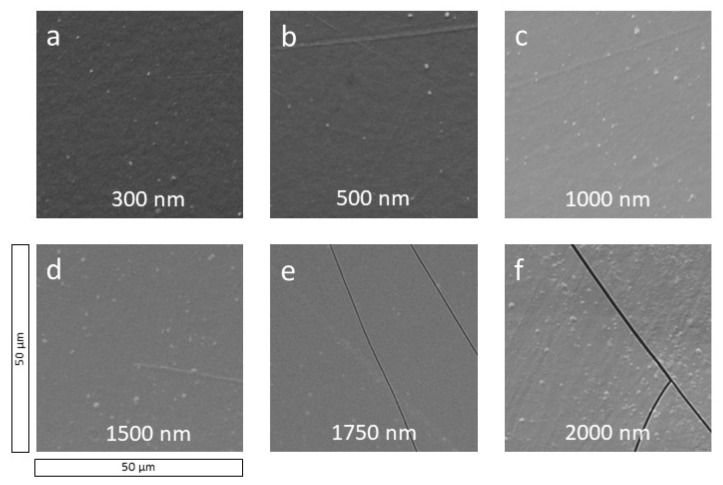
SEM analysis of thick a-C:H layers on PA6 (**a**–**f**). Thickness varies from (**a**) 300 to (**f**) 2000 nm, arranged in increasing layer thickness.

First, SEM images are discussed to get an overview of the layer condition. Figure 1 shows the reference sample (Figure 1a), the O_2_ plasma-treated sample (Figure 1b), and a-C:H coated samples up to a layer thickness of 100 nm (Figure 1c–l). Thicker layers are displayed in Figure 2.

The reference sample of PA6 (Figure 1a) is strongly structured and shows many scratches, which are due to the production process. Additionally, some minor impurities in the form of particles are visible. The O_2_ plasma-treated sample (Figure 1b) is smoother and has fewer scratches. In addition, there are less dirt particles on the surface. With the application of a-C:H layers of increasing thickness starting at 10 nm (Figure 1c–l) the scratches become weaker as they are covered with the a-C:H layer. The deposited layer is closed and appears homogeneous. On the 50 nm sample (Figure 1g) three black stripes are visible. Here, the gold layer applied for the SEM measurements is probably damaged, which leads to charging effects and thus to a darker appearance. With increasing thickness, the scratches of the base material are still visible, but the layer appears closed overall and adheres well.

In Figure 2a–f, the up to 2 µm thick a-C:H layers deposited on PA6 are shown. With 300 nm (Figure 2a) the surface of the layer is homogeneous and closed. The scratches are further attenuated. This behavior continues up to 1500 nm (Figure 2b–d), but when the a-C:H layer reaches 1750 nm (Figure 2e), the layer breaks up. The layer failure continues with the PA6 sample coated with 2000 nm (Figure 2f).

**Figure 3:**
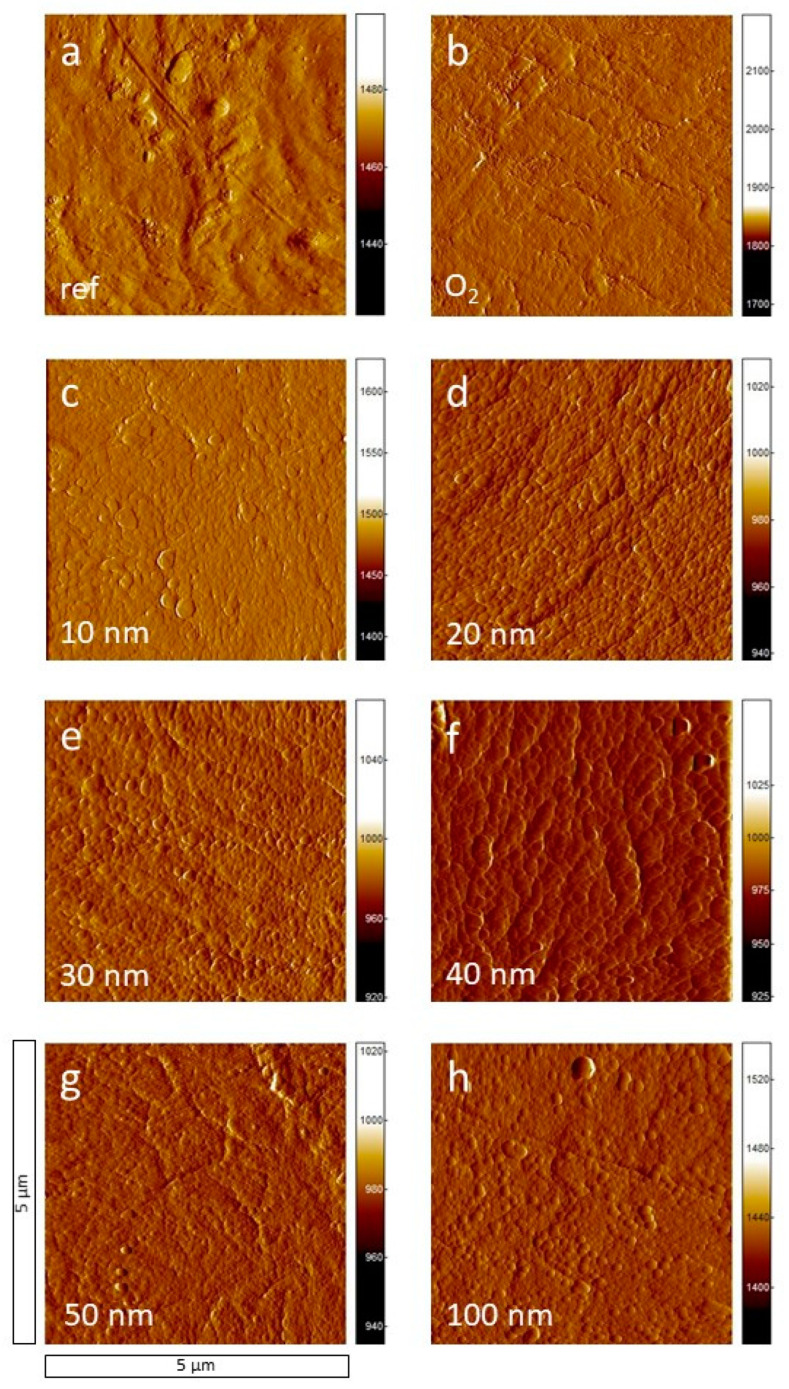
AFM images of the raw PA6 (**a**), the O_2_ plasma-treated PA6 (**b**) samples, and PA6 samples coated with thin a-C:H layers of various thickness (10 to 100 nm, **c**–**h**). The image size is 5 μm × 5 μm.

For a detailed investigation of the surface morphology, high-resolution AFM measurements were performed. Figure 3a shows the surface morphology of raw PA6 (reference sample), the PA6 cleaned with oxygen plasma (Figure 3b), and PA6 covered with a-C:H layers from 10 to 100 nm (Figure 3c–h) in ascending order. The measured area has a size of 5 µm × 5 µm, the roughness values for the AFM measurements (raw PA6 to 2000 nm a-C:H@PA6) are shown in Table 1. The roughness *R*_q_ is in the interval of 5.12 ± 0.55 nm (1000 nm deposition) and 9.20 ± 0.11 nm (100 nm deposition) for all samples measured resulting in a relatively smooth layer. For the raw and the O_2_ plasma-treated PA6 sample, the surface is bumpy and shows some scratches, which could be a result of the manufacturing process. Some particles which are visible on the AFM image can be dust or dirt. These are unavoidable due to the industrial production of the samples and the prevalent environment there. For the AFM images, the surface of the oxygen-treated sample (Figure 3b) appears to be a little more roughened compared to the raw material (*R*_q_ of 6.81 ± 1.10 nm compared to 6.21 ± 0.68 nm), which is most likely caused by the oxygen bombardment. Additionally, some scratches are still visible. With the deposition of a 10 nm a-C:H layer on PA6 (Figure 3c), the surface is covered by small, unstructured, grain-like particles resulting in a raised *R*_q_ value of 8.22 ± 1.31 nm. This more uneven morphology of the coating decreases with increasing a-C:H layer thickness and an overall more even layer is formed, which has a defined grain-like morphology. This starts with a thickness of 40 nm. The beginning growth of a homogeneous grain structure resp. layer represents the completion of the interlayer phase [14]. Here, the roughness is 9.04 ± 0.92 nm and nearly at its maximum value. The interlayer is a mixed phase of polymer and a-C:H layer, whose thickness can be estimated to 40 nm by the current AFM investigations. The start of the normal layer growth at 50 nm a-C:H thickness is accompanied by a lower *R*_q_ (8.73 ± 0.58 nm). The scratches already visible on raw PA6 are still visible on thinner layers, especially on a-C:H layers of 20, 30, and 100 nm (Figure 3d,e,h). This behavior is similar to that of such a-C:H layers on high-density polyethylene (HDPE), which have already been investigated by Catena et al. [14].

**Figure 4:**
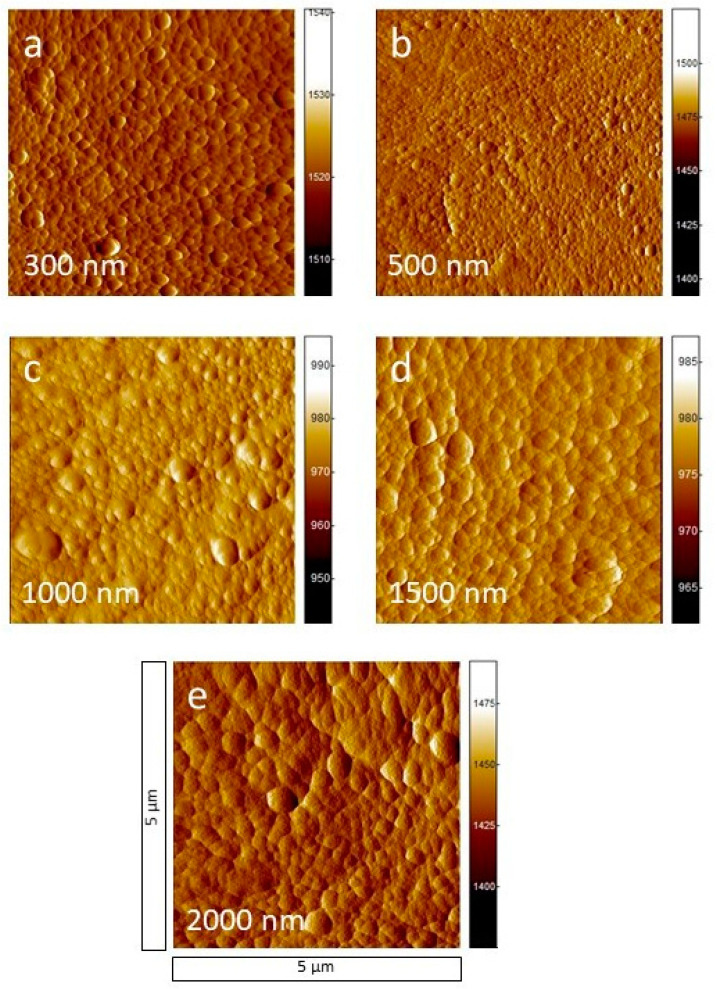
AFM measurements for PA6 samples (**a**–**e**) coated with thick a-C:H layers from (**a**) 300 up to (**e**) 2000 nm. The image size is 5 μm × 5 μm.

Figure 4 shows the AFM images of the deposition of thicker a-C:H layers from 300 up to 2000 nm. The homogeneous grain structure, which became observable for the thin layers beginning at 40 nm, continues for the 300 nm layer (Figure 4a). With a thickness of 500 nm (Figure 4b) the majority of the grains becomes smaller compared to the grains of the 300 nm layer (*R*_q_ of 6.14 ± 0.91 nm compared to 6.74 ± 0.27 nm). This may be a consequence of a new grain layer starting on top of the previous closed one at a lower thickness. The AFM images for 1000, 1500, and 2000 nm (Figure 4c–e) show smoother grains than before, resulting in the lowest surface roughness of 5.12 ± 0.55 nm at 2000 nm a-C:H layer thickness. They become more regularly spaced, but differences in grain size remain constant. The AFM images confirm the homogeneous surface coating of the PA6 samples with a-C:H for higher layer thicknesses, as already indicated by the SEM (Figure 2). For thin layers, it could also be determined that this homogeneous layer growth starts only at about 40 nm with the completion of the interlayer. This could not be detected on the SEM due to its lower resolution. However, with SEM it was possible to show the layer failure at higher layer thicknesses which, of course, is poorly detectable in AFM. Here, both techniques complement each other well.

CA measurements were performed for further analysis of the surface characteristics. The wettability of the surface and thus the resulting CAs of the investigated a-C:H layers are influenced by three factors: (I) the morphology of the surface [36,37,38]; (II) the different carbon hybridization states on the surface [18,21,39,40,41]; and (III) the existing chemical bonds [42,43,44,45]. A detailed description is summarized in a previous paper by some of the authors [26].

**Figure 5:**
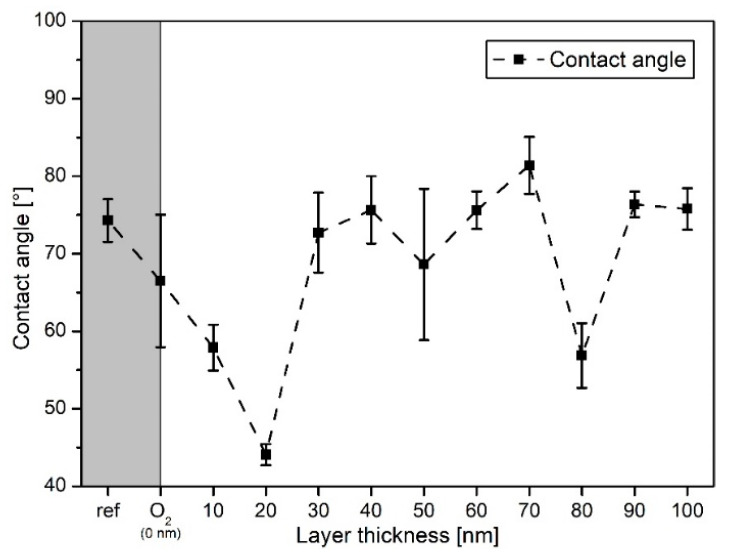
Contact angle measurements for the PA6 samples: shown are raw and O_2_ plasma-treated PA6 as well as thin a-C:H layers up to 100 nm (the dashed line only indicates the trend).

For a better overview, the CA evaluation is divided into two diagrams. Figure 5 shows the CAs obtained for the series of a-C:H layers from 10 to 100 nm deposited on PA6. Additionally, the CA of the raw and e O_2_ plasma-cleaned PA6 are shown. The plasma-cleaned sample is used here as the reference since all a-C:H deposited samples were previously O_2_ plasma cleaned. For the raw PA6 polymer the CA is 74° and within the limits of the literature values [46]. With O_2_ plasma treatment, the angle decreases to 66°. As discussed earlier, this is due to the presence of functional oxygen groups. The surface becomes more hydrophilic and the CA decreases. With the application of 10 nm a-C:H layer, the CA drops rapidly to 58°. This behavior continues until the 20 nm deposition, where the lowest measured value of 44° is reached for the present series. If the film thickness is increased to 30 nm, the CA changes to 72°. Up to a layer thickness of 60 nm, the CA varies between 68° and 76°, meaning it remains relatively constant within the error margins, even for the relatively large error at 50 nm. This also indicates the completion of the interlayer phase in the 30 to 40 nm region, since a new growth phase starts. With a layer thickness of 80 nm the CA decreases again sharply to 57° before returning to 75° at 90 nm. In summary, for the thin film depositions (10 to 100 nm, Figure 5) according to the CAs, an overall similar film is obtained, with few exceptions only after reaching 30 nm, despite continued growth resp. increase of layer thickness. For 30 to 100 nm a-C:H and the raw PA6, with the exception of 80 nm deposition, the CA values are comparatively high, which is associated with a sp^2^-like carbon hybridization. The CA of the 10, 20, and 80 nm depositions are below 60°, which indicates a more sp^3^-like hybridization of the carbon atoms.

The gray box depicts values for the raw polymer and the O_2_ plasma-pretreated sample. The layer thickness of 0 nm is depicted by the O_2_ sample.

**Figure 6:**
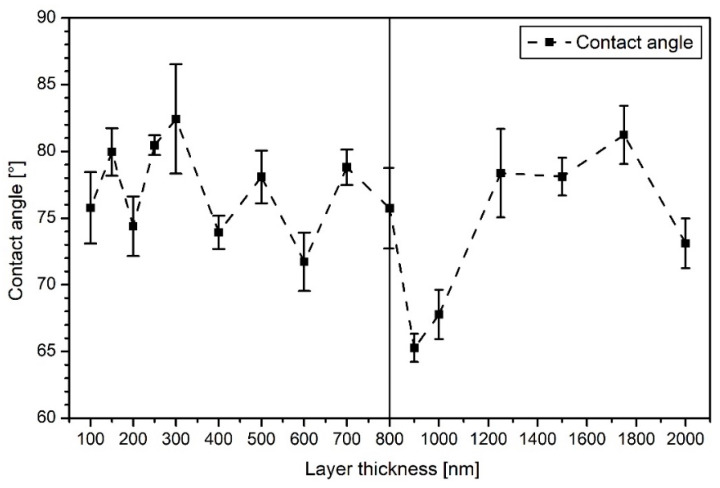
Contact angles of amorphous hydrogenated carbon (a-C:H) coatings of layer thicknesses from 100 to 2000 nm on PA6 samples (the dashed line only indicates a trend).

Figure 6 shows the CA measurements for the thicker a-C:H depositions from 100 to 2000 nm on PA6. For the layers between 100 and 800 nm, the CAs alternate between 75° and 82°. With reaching a layer thickness of 900 nm, the CA decreases to 65°, but recovers at 1250 nm within 78° and 81° until 1750 nm. At 2000 nm, the CA decreases again. The temporarily strong decrease in CAs between 800 and 1250 nm may indicate a change of the hybridization state to a more sp^3^-dominated network. It is also conceivable that dehydrogenation of the layer occurs right after, resulting in a higher contact angle due to the higher amount of sp^2^-bound carbon, i.e., a reduced proportion of hydrogen in the bonds present. This has already been proven for HDPE in this a-C:H thickness range [14,23]. The error of the CA measurements is relatively small in most cases, which suggests a flat, homogeneous surface, as already visible in SEM (Figure 1 and Figure 2) and AFM (Figure 3 and Figure 4). For the layers above 1750 nm, which were visibly broken in SEM (Figure 2e,f), a larger error was to be expected due to the non-closed surface. The relatively small error is possibly due to the limited amount of cracks. A change in the bonding ratio of the carbon atoms and the CAs, as in other materials before, could not be observed [24,25].

### 3.2. Infrared Spectroscopy

**Figure 7:**
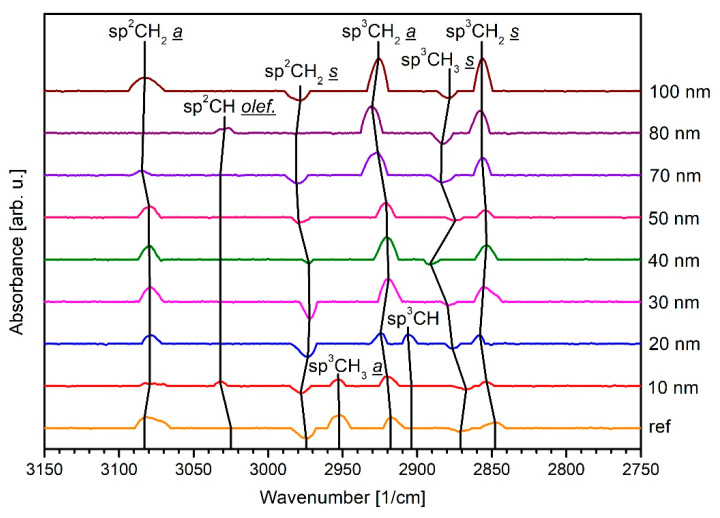
DRIFT spectra of the analyzed PA6 samples coated with thin a-C:H up to 100 nm in 10 nm steps starting from the O_2_ plasma-treated reference. The measurement series is applied from below to above with increasing layer thickness.

**Figure 8:**
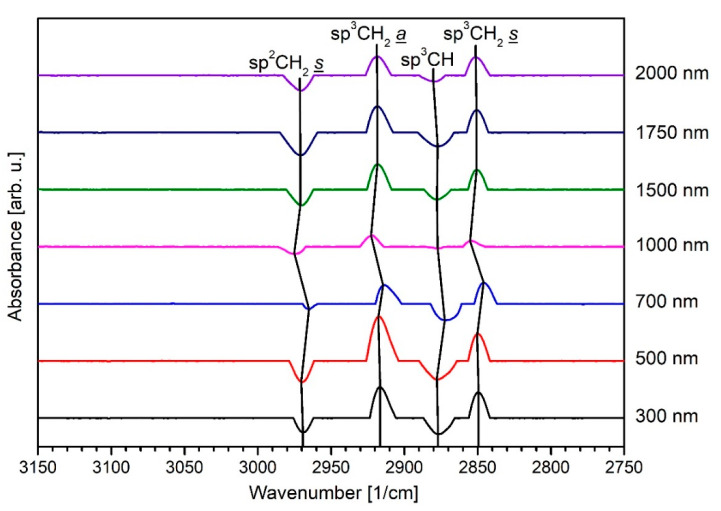
DRIFT spectra for PA6 samples coated with thick a-C:H from 300 to 2000 nm with increasing layer thickness from below.

DRIFT spectroscopy allows examining differences in the chemical composition of the a-C:H layers deposited on the PA6 samples as a function of their specific thickness. In particular, the measurements presented here provide information on the various sp^2^ and sp^3^ bonds of the carbon atoms present in the layers. The results of the DRIFT spectra in Figure 7 and Figure 8 are evaluated on the basis of own results and those of other groups [15,16,47,48]. The O_2_ plasma-treated PA6 is used as a reference (“background”) for the samples investigated here since all a-C:H layers are deposited on a previously oxygen-treated PA6 surface.

Figure 7 and Figure 8 show the corresponding spectra in ascending order plotted in arbitrary units (arb. u.). For a better overview, the obtained results are shown separately: the raw PA6 sample and the thinner a-C:H layers from 10 to 100 nm are plotted in Figure 7, the thicker layers from 300 to 2000 nm in Figure 8. Thinner layers were coated and measured in 10 nm steps due to special interest in the interlayer formation taking place in this region. Figure 7 shows that a total of eight different oscillations of carbon bonds occur for the thin layers. It is noticeable that the peak orientation according to the PA6 sample treated with O_2_ plasma contains more sp^3^ (sp^3^CH_3_ *a* = asymmetric at 2952 cm^−1^, sp^3^CH_2_ *a* is at 2917 cm^−1^, and sp^3^CH_2_ *s* = symmetric at 2847 cm^−1^ [47,48]) to both the raw polymer and all a-C:H layers. The lower proportion of sp^3^ bonds on the surface of the raw polymer is due to impurities that have settled there and been removed by the oxygen plasma. In addition, only two of the bonds measured are absorption oscillations: the comparatively dominant sp^2^CH_2_ *s* oscillation at 2974 cm^−1^ and the sp^3^CH_3_ *s* at 2871 cm^−1^ [48], which is relatively weak. For the layers, this means that the carbon is mostly sp^2^ bounded. Vibrations for sp^2^CH olefinic (3032 cm^−1^ [48]) are only visible for the 10 and 80 nm a-C:H layer, and they are comparatively weak and will not be further discussed.

The sp^3^CH_3_ *a* peak is also not discussed further, as it is only available for the reference sample and the 10 nm a-C:H coating. Since this layer thickness corresponds approximately to the measuring depth, signals of the PA6 material are transmitted. In the course of the current coating process, the surface of the a-C:H layer undergoes some chemical changes, which are characterized by peaks of varying intensity. Overall, however, the same bond oscillations are observed for all measured layer thicknesses. A noticeable change as function of film thickness is a significant shift in the peaks, i.e., a slight change of the peak positions. A shift to a larger wave number means, here, a higher C–H binding energy. This is expressed by a smaller binding distance between the C–H atoms [32]. The sp^2^CH_2_ *a* bond shifts to a higher wavenumber at a layer thickness of 80 nm. The sp^2^CH_2_ *s* bond, in contrast, undergoes several shifts. Firstly, during layer growth to 10 nm to higher wavenumbers, and then returning to the old position when changing to 20 nm. After reaching a layer thickness of 40 nm, the peak shifts again to higher wavenumbers. The sp^3^ bonds carry out a large number of shifts and change minimally at almost any layer thickness. Changing binding distances, recognizable by frequencies shifting back, indicate a changing in chemical composition. Thus, a chemically very variable environment is present on the surface with sp^2^-dominated absorption behavior. A large number of well-formed sp^3^ peaks, which are not absorption peaks, indicate a lower sp^3^ content compared to the O_2_ plasma-treated sample and support the statement of sp^2^ dominated growth for the first 100 nm layer thickness. A similar behavior has been observed for such a-C:H coatings on other polymers [15,16,23,24,25,26].

Figure 8 shows the DRIFT measurements for the thick layers greater than 100 up to 2000 nm layer thickness. For thicker a-C:H layers on PA6, most of the peaks previously visible for thin layers disappear (compare Figure 7). A total of four oscillations of carbon bonds can be identified. As absorption peaks, the sp^2^CH_2_ *s* and the sp^3^CH peak as well as the oscillations of the symmetric and asymmetric sp^3^CH_2_ bond are also visible. These indicate their presence in the O_2_-treated sample, but not or only to a relatively small extent in the a-C:H layers. As with the thin layers, all bonds undergo a large number of position shifts, so the surface is chemically very variable. For 300 and 500 nm, the peaks for the sp^2^ and sp^3^ oscillation are both well defined. With a change to 700 nm film thickness, the sp^3^CH bond dominates the absorption peaks. This indicates that with increasing a-C:H layer thickness, more sp^3^ bonds are formed. These bindings are very strong in comparison to sp^2^ and sp bonds, due to their tetrahedral shape [21]. When the 1500 nm thickness is reached, the sp^3^ bond weakens and the sp^2^ bond becomes dominant until 2000 nm. Additionally, with reaching the 1500 nm a-C:H, all peaks shift back to their values from the beginning. This is an indication of the dehydrogenation of the layer. Hydrogen is released from the carbon sp^3^ bonds by conversion to sp^2^ bonds, whose proportion increases. This is in line with the results of the CA measurements which is rising in this film thickness region. Dehydrogenation could already be observed for HDPE for a similar thickness range [14,23].

### 3.3. X-ray Spectroscopy

**Figure 9:**
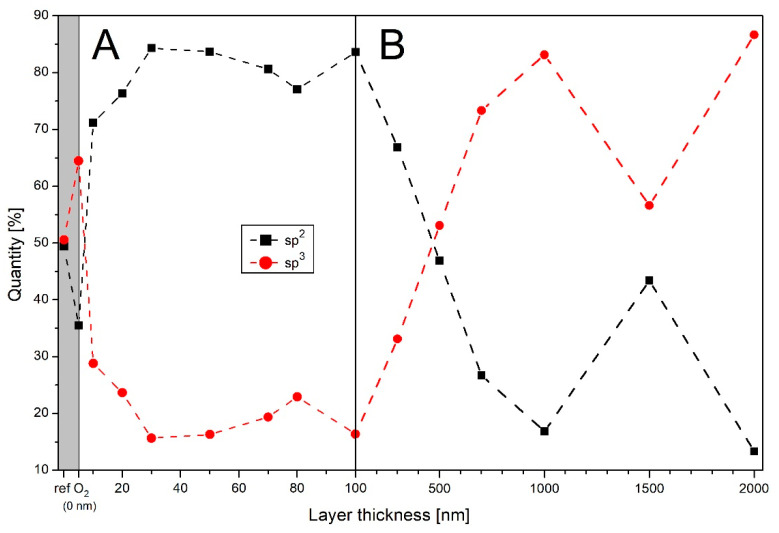
XPS measurements for PA6 coated with a-C:H layers of various thickness. The content of the sp^2^ (black squares) and sp^3^ (red circles) bound carbon are plotted against layer thickness: (**A**) thin layers from raw PA6 (ref) and O_2_-cleaned material (represents a layer thickness of 0 nm) to 100 nm and (**B**) thicker layers up to 2000 nm (the dashed line only indicates a trend). The gray box indicates the region before a-C:H coating (0 nm layer thickness).

For further analysis of the chemical composition of the deposited a-C:H layers on PA6, measurements were performed using the X-ray synchrotron-based techniques XPS and NEXAFS. Figure 9 shows the evaluated results for the XPS measurements (for deconvolution results, see Supplementary Material). Here, the sp^2^ and sp^3^ bonds are plotted as a function of film thickness. Peak positions were identified using the NIST database and other publications [24,25,49,50,51]. For a better overview, the graph has been separated into Figure 9A for thin films from 0 to 100 nm (including the raw PA6 and O_2_ plasma-treated sample) and Figure 9B for thick films from 100 to 2000 nm. A more detailed analysis of the thinner layers was carried out to investigate the formation of the interlayer, which is a mixture of base polymer and the a-C:H layer.

The existence of the interlayer has already been proven for other polymers such as polyethylene terephthalate (PET) and HDPE [14,15,23,28], and in the case of HDPE its thickness has also been determined by X-ray spectroscopic analysis methods and DRIFT measurements [23]. For the raw polymer, about 50% sp^2^- and 50% sp^3^-bound carbon were determined (Figure 9A). Considering the structure of the raw polymer, an almost pure sp^3^ structure is expected and the raw polymer is obviously dirty. This may be from residues resp. contaminants of the manufacturing process or softeners detaching from the polymer over time. The contamination was already detectable in the DRIFT measurements, where pronounced sp^2^ oscillations are visible (Figure 7). With the O_2_ plasma treatment, the amount of sp^2^ bonds present was strongly reduced and the surface was cleaned and activated. For this reason, all samples coated later on were first O_2_ plasma treated. With the application of a 10 nm thick a-C:H layer, the proportion of sp^2^ bonds increases again at the cost of decreasing sp^3^ bonds. This trend continues with increasing layer thickness until a thickness of 30 nm is reached. Here, the sp^3^ bonds have the lowest proportion and the layer composition remains almost constant up to 50 nm. The increased sp^3^ content up to 30 nm is probably due to fragments of the base polymer, which are deposited in the interlayer phase with layer components. With increasing layer thickness, their percentage in the interlayer decreases, since the base material is no longer available due to possible erosion processes. The interlayer phase ends with a layer thickness of about 30 nm and pure a-C:H growth begins. After reaching 50 nm film thickness, the sp^3^ content increases again but reaches only 23% at 80 nm. At 100 nm, this value is again 17%. From the completion of the interlayer to 100 nm layer thickness, the value of sp^2^- and sp^3^-bound carbon is almost constant, even if there are small fluctuations. The dominance of sp^2^ bonds and decreasing proportion of sp^3^ bonds, which are visible in XPS, were already seen in the DRIFT measurements. The CA measurements also indicate a strong sp^2^ dominance for thin films, and the increase in sp^3^ bonds at 80 nm is also visible there. Therefore, all techniques reveal a very similar behavior.

Figure 9B shows the thicker layers from 100 to 2000 nm. If the layer thickness increases, the bonding ratio at the surface of the a-C:H layer starts to change. The proportion of sp^3^ bonds increases strongly, and at 500 nm the bond ratio changes to sp^3^-dominated a-C:H growth. At 1000 nm, about 83% of the carbon is sp^3^ bound at the surface. With the step to 1500 nm, the proportion of sp^2^ bonds increases strongly again to 43%. This is due to the dehydrogenation of the layer. Hydrogen leaves the layer at the expense of the sp^3^ bonds. This leads to a re-hybridization of the carbon atom orbitals. The now free bonds of the previously sp^3^-bound carbon changes into sp^2^-hybridized carbon clusters. The decrease in sp^3^ bonds in favor of increasing sp^2^ bonds was already visible in the DRIFT (Figure 8) starting at 1500 nm. Moreover, the coalescence phenomenon of the fusion of grains on the surface takes place in this region, as demonstrated by some authors [14]. This behavior has already been demonstrated for HDPE [14,23]. If the thickness of the layer increases again, the effect decreases, and carbon is again strongly sp^3^ bound at 2000 nm. The XPS results clearly show that layer growth up to 100 nm is sp^2^ dominated and towards thicker layers a more rigid, sp^3^-dominated carbon network is present on the surface. Exceptions are the interlayer region, which is limited to the first 30 nm layer thickness and the zone of dehydrogenation at 1500 nm a-C:H layer.

**Figure 10:**
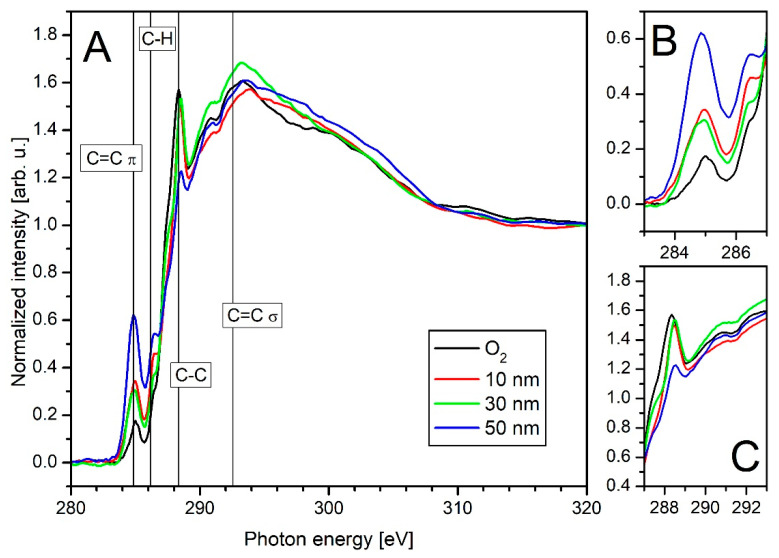
C K-edge NEXAFS measurements for PA6 coated with thin layers of a-C:H: (**A**) shows the full spectra; (**B**,**C**) present detailed photon energy intervals for carbon relevant peaks. (**B**): 283.00 to 287.00 eV for the C=C π peak (284.85 eV) and the C–H peak (286.15 eV); (**C**): 287.00 to 293.00 eV for the C–C and C=C σ (288.15 eV and 292.58 eV).

For thin layers, additional NEXAFS measurements have been performed. The spectra of the O_2_ plasma-treated PA6 together with various thin a-C:H layers on PA6 (10, 30, and 50 nm) are shown in Figure 10. The recording and evaluation of the raw data are performed as described earlier in the Section 2.4 experimental section (2.4). In consideration of the work of other groups, the peak positions were determined as follows: C=C π (284.85 eV), C–H (286.15 eV), C–C (288.35 eV), and C=C σ (292.55 eV) [52,53,54,55,56]. For a better overview, the figure is divided in three areas: A for the full spectra of the carbon C K-edge from 280–320 eV, B for the area of 293–287 eV for the C=C π, and C from 287–293 eV for the C–C binding.

For the O_2_ plasma-treated sample (black curve) it is noticeable that the C=C π peak belonging to the sp^2^ is only very weakly pronounced at 284.85 eV, while the C–C peak, which represents the sp^3^ bonds, is strongly emphasized. This corresponds to the previous DRIFT and XPS results. With the application of a 10 nm a-C:H layer (red curve), the sp^2^ fraction increases, but the sp^3^ fraction remains almost unchanged. In addition to the sp^3^ bonds (C–C peak) of the substrate, there are also sp^2^ bonds (C=C π peak) detectable due to the deposited layer. If the layer thickness is increased to 30 nm (green curve), a spectrum similar to that of the 10 nm sample is obtained. In both curves, the sp^3^ content most likely comes from the base material and is part of the interlayer. However, if the layer thickness of 50 nm is reached (blue curve), the measured spectrum changes significantly. The C=C π increases strongly while the C–C peak almost disappears. The reason for the disappearance of the C–C peak is that it was caused by the base material which was mixed with the a-C:H layer in the interlayer. At 50 nm layer thickness, only the pure a-C:H layer is measured. The end of the interlayer phase is therefore specified between 30 and 50 nm thickness, which was already shown in the AFM, CA, DRIFT, and XPS measurements.

## 4. Conclusions

Polymer PA6 was coated with a-C:H layers in various thicknesses (10–2000 nm) using RF-PECVD generated acetylene plasma. The a-C:H layers were analyzed in two separate ranges: thin ones up to 100 nm to investigate the interlayer phase and thick layers between 100 and 2000 nm to investigate the dehydrogenation. The SEM images show stable a-C:H layers on PA6 samples, provided that the layer thickness of 1500 nm is not exceeded. Here, dehydrogenation also takes place, leading to a decrease in sp^3^ bonds in favor of sp^2^ bonds. The layer failure can be attributed to a stress reduction due to the sp^3^ dominance still present in this region.

AFM, CA, DRIFT, and XPS measurements show that the interlayer formation for a-C:H films on PA6 is completed by 40 nm. In the AFM measurements, a homogeneous grain structure can be seen from here on. In the CAs, a relatively constant angle for the thin films is obtained after 40 nm. In XPS, the sp^3^ content, which is lifted from the raw material into the interlayer mixed phase, is almost constant and low and a pure, sp^2^ containing a-C:H layer grows up. This behavior changes only after the layer thickness exceeds 100 nm and the carbon in the layer begins to be more strongly sp^3^ bound. The SEM and AFM measurements show equally stable growth. In the further course, the sp^3^ content increases significantly until dehydrogenation takes place at 1500 nm and stress-induced layer failure occurs later on.

Furthermore, it can be seen that the bonding ratios of the carbon atoms depend not only on the selected plasma parameters, but also on the achieved layer thickness, which clearly underlines the changing character of the a-C:H layer with increasing thickness. Both DRIFT and XPS measurements show a change between sp^3^- and sp^2^-dominant bound layers. Since the change in bond dominance also causes a change in the properties of the applied layer, these can be controlled accordingly and adjusted by the layer thickness. The thickness and the chemical environment of the interlayer between PA6 and the a-C:H layer could also be determined, very accurately, to 40 nm. This value is therefore very pronounced and also explains the high stability of the applied a-C:H layer before it breaks up at 1750 nm. The dehydrogenation of the layer at high thicknesses, which is also known for other coated polymers, could also be proven.

In the process of the studies, it was shown that a-C:H layers can be deposited successfully on the PA6 material and remain stable (= without cracks) up to a thickness of 1500 nm. The layer also adheres properly to the polymer (interlayer = 40 nm). It is therefore possible to refine PA6 for applications such as in the (food) packaging sector (e.g., changed barrier effects).

## Figures and Tables

**Table 1:** AFM roughness values *R*_a_ and *R*_q_ for the reference, the O_2_ plasma-treated, and with PA6 samples coated with thin a-C:H layers of various thickness.

Sample	*R*_a_ [nm]	*R*_q_ [nm]
Reference	4.74 ± 0.37	6.21 ± 0.68
O_2_	5.25 ± 0.76	6.81 ± 1.10
10 nm	6.25 ± 1.05	8.22 ± 1.31
20 nm	6.52 ± 0.25	8.25 ± 0.40
30 nm	5.98 ± 0.04	7.61 ± 0.27
40 nm	6.86 ± 0.49	9.04 ± 0.92
50 nm	6.53 ± 0.44	8.73 ± 0.58
100 nm	6.73 ± 0.02	9.20 ± 0.11
300 nm	5.15 ± 0.11	6.74 ± 0.27
500 nm	4.81 ± 0.62	6.14 ± 0.91
1000 nm	4.86 ± 0.12	6.28 ± 0.18
1500 nm	5.09 ± 1.90	6.63 ± 2.12
2000 nm	3.98 ± 0.29	5.12 ± 0.55

## Data Availability

The data presented in this study are available on request from the corresponding author.

## Supplementary Materials

The following are available online at https://www.mdpi.com/article/10.3390/polym13111886/s1, Figure S1: XPS C1s Peak deconvolution for the reference, O_2_ plasma treated sample and the thin layers from 10–100 nm a-C:H film thickness, Figure S2: XPS C1s Peak deconvolution for the PA6 samples coated with thick a-C:H layers (300–2000 nm).
