# Supplementary material for: Plasma Supported Deposition of Amorphous Hydrogenated Carbon (a-C:H) on Polyamide 6: Determining Interlayer Completion and Dehydrogenation Effects during Layer Growth
Source: Polymers (Basel). 2021 Jun 6;13(11):1886. doi: 10.3390/polym13111886 (PMC8201342; doi:10.3390/polym13111886)
Supplement: Supplementary file 1 [file polymers-13-01886-s001.zip › SI-polymers-1219604-XPS-all-layers-Rev.pdf]

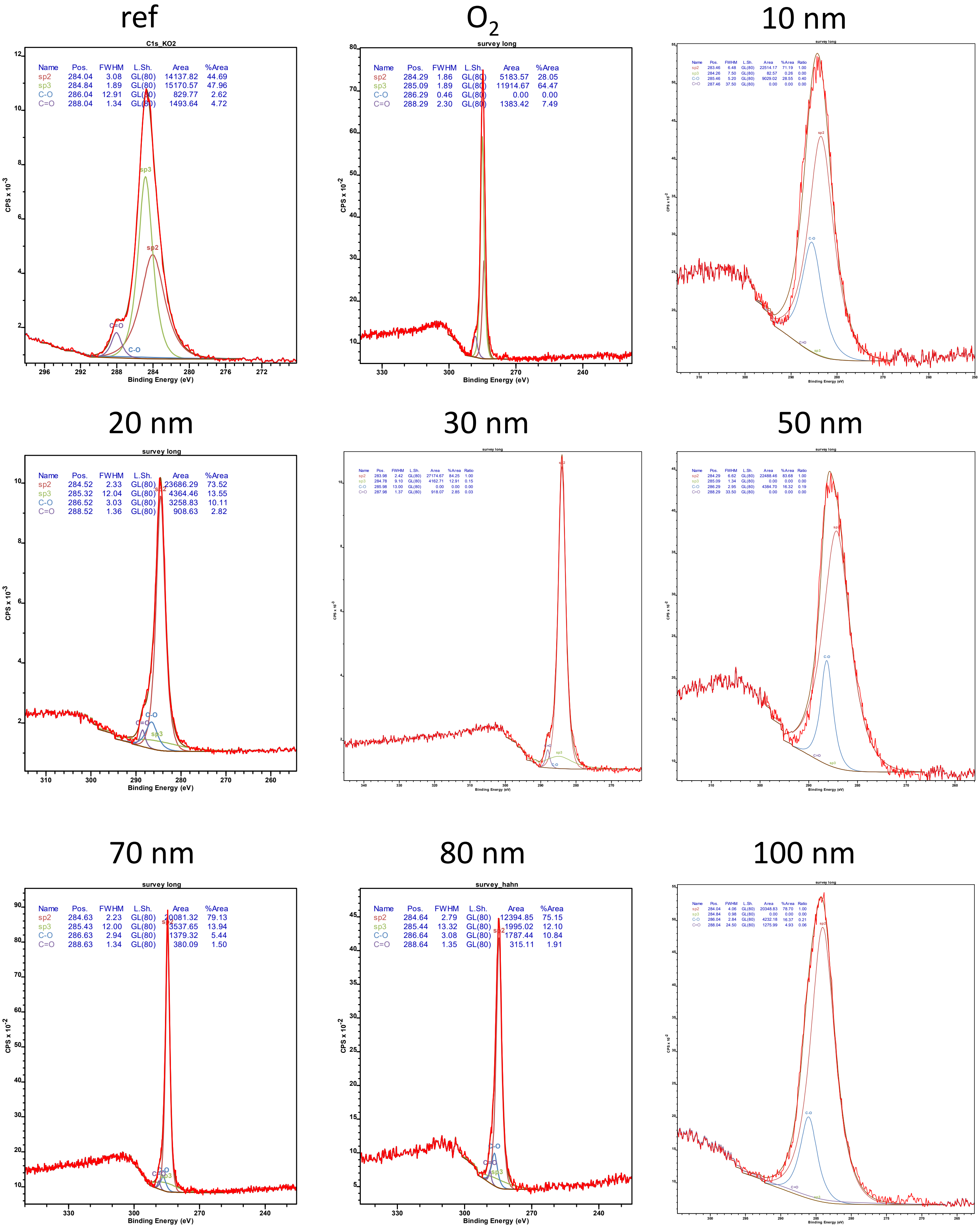

SI 1. XPS C1s Peak deconvolution for the reference, O_2 plasma treated sample and the thin layers from 10-100 nm a-C:H film thickness.

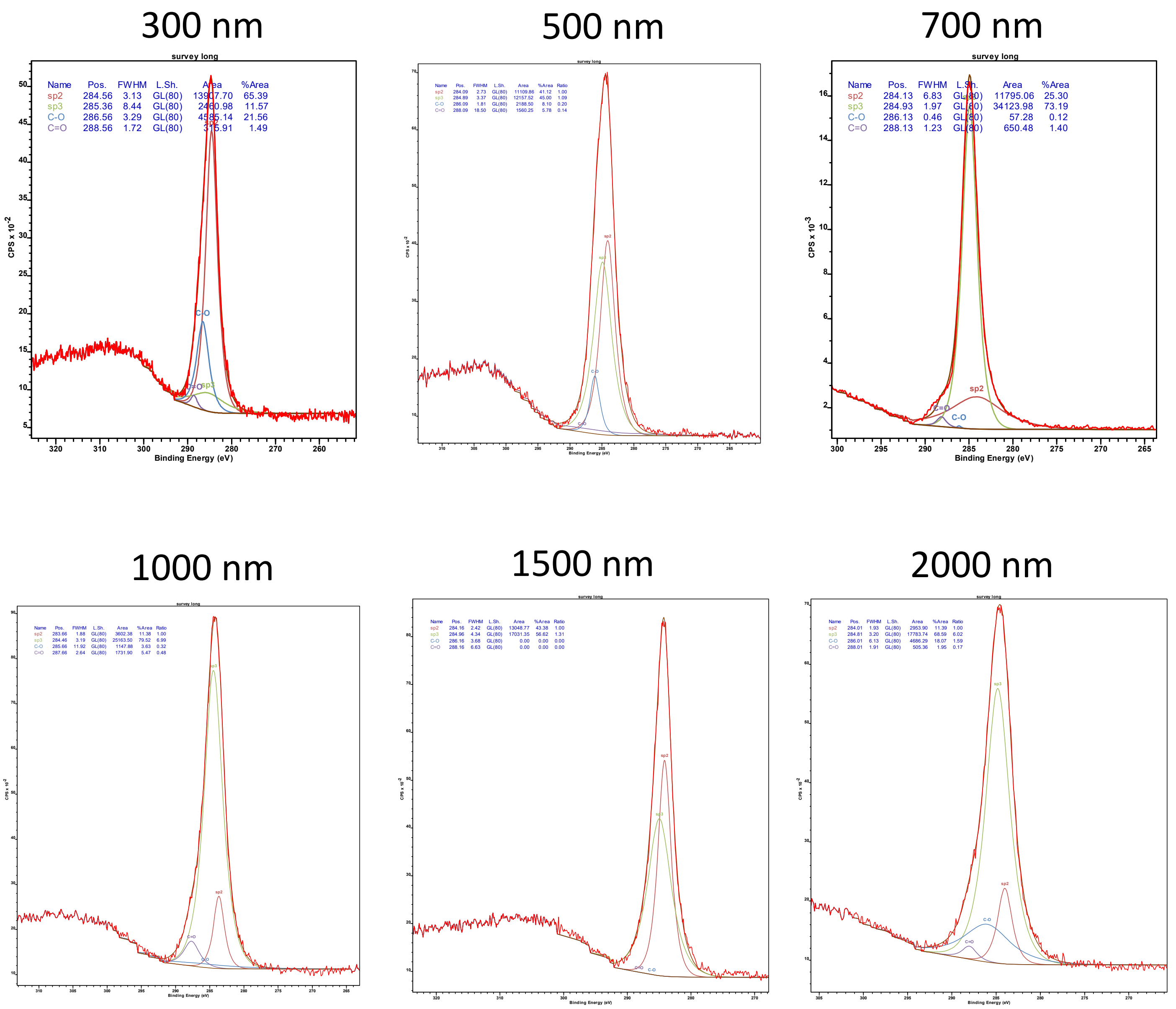

SI 2. XPS C1s Peak deconvolution for the PA6 samples coated with thick a-C:H layers (300-2000 nm).
